# Drug-related stigma among people who inject drugs – development and validation of the drug use stigma scale (DUSS)

**DOI:** 10.1371/journal.pone.0338691

**Published:** 2025-12-12

**Authors:** Robin A. Pollini, Catherine E. Paquette, Brandon Irvin, Jennifer L. Syvertsen, Christa L. Lilly

**Affiliations:** 1 Department of Behavioral Medicine & Psychiatry, West Virginia University, Morgantown, West Virginia, United States of America; 2 Department of Population Health Sciences, Duke University, Durham, North Carolina, United States of America; 3 Department of Anthropology, University of California Riverside, Riverside, California, United States of America; 4 Department of Epidemiology & Biostatistics, West Virginia University, Morgantown, West Virginia, United States of America; Johns Hopkins University Bloomberg School of Public Health, UNITED STATES OF AMERICA

## Abstract

Drug use is a highly stigmatized behavior, and drug-related stigma is a key driver of behavioral risk, lower health care utilization, and associated adverse health outcomes among people who inject drugs (PWID). While instruments exist for measuring drug-related stigma, their applicability to community-based PWID across multiple stigma types (enacted, anticipated, internalized) and settings (health care, society, family) is limited, as most were developed using treatment-based samples and all were developed in urban populations. This study sought to develop a Drug Use Stigma Scale (DUSS) that addresses these limitations. We developed an initial list of 39 items based on literature review and qualitative interviews (N = 27) and three focus groups (N = 28) with PWID recruited from syringe services programs and via peer referral in two predominantly rural West Virginia counties. The scale items were administered in a survey to 336 PWID recruited from the same two counties divided into development and validation samples. Responses to the 39-item scale went through a multidimensional refinement process, including examination of internal consistency, Confirmatory Factor Analysis (CFA), and a three-factor CFA based on stigma setting. Next, a set of final measurement CFAs were conducted. Finally, the resulting scale was examined for criterion-related concurrent validation. The final DUSS consisted of 16 items with excellent fit statistics for the development sample: SRMR: 0.03, RMSEA: 0.09, GFI: 0.92, CFI: 0.96, NFI: 0.94. Fit attenuated but remained satisfactory for the validation sample. DUSS scores were significantly associated with increased odds of not seeking healthcare when needed (OR: 1.47, p = 0.001; OR: 1.61, p < 0.0001) after controlling for gender and age. Thus, the resulting scale, the DUSS, demonstrates strong validity and internal reliability across multiple stigma mechanisms and settings in a rural population, making it a valuable tool for broadly assessing drug-related stigma and related intervention effectiveness among PWID.

## Introduction

Drug use is a highly stigmatized behavior, and people who inject drugs (PWID) experience even greater stigma than those who use drugs through non-injection routes [1,2]. Research suggests that drug-related stigma in healthcare and social settings is a key driver of behavioral risk, lower healthcare utilization, and associated adverse health outcomes among PWID [3–7]. These include studies linking stigma to high risk injection practices [8], drug overdose [9,10], lower uptake of HIV prevention services [7,11–17], and delayed medical care (e.g., for injection-related infections) [4,6,7,18–21]. Experiences of drug-related stigma are also associated with poor mental health [10,22], including lower self-esteem and greater depression, anxiety, and suicidal ideation [23–26]. As the evidence supporting the adverse impacts of drug-related stigma has grown, so too have the calls for effective stigma reduction interventions [27–29]. Despite these findings and calls for action, PWID have not been centered in efforts to develop valid and reliable measures of drug-related stigma.

The literature on drug-related stigma highlights the challenges inherent in measuring a concept as complex as stigma, which Erving Goffman broadly defined as “an attribute that is deeply discrediting.” [30]. Existing peer-reviewed research on substance use stigma is largely qualitative and draws from multiple theoretical frameworks. The literature describes key mechanisms through which people who use drugs experience stigma; these include enacted stigma (experiences of unfair treatment or discrimination due to association with a stigmatized group), anticipated stigma (the expectation of being discriminated against or treated poorly because of a stigmatized identity), and internalized stigma (adopting negative beliefs and attitudes about oneself based on a stigmatized identity) [31,32]. These categorizations were derived from a broad and interdisciplinary body of stigma-related literature. For example, a review of previous research on stigma theory identified the mechanisms by which individuals with HIV encounter stigma; the resulting HIV Stigma Framework [33,34] outlined the categories of enacted, anticipated, and internalized stigma that have now been widely applied to substance use stigma as well [32].

Quantitative efforts to develop a drug-related stigma scale applicable to U.S. PWID have a variety of limitations. Most rely on in-treatment samples, which represent only a small percentage of people who use drugs in the U.S. and may include individuals with alcohol use disorders who do not use criminalized drugs [2,35–38]. Some scales limit their focus to a certain type of stigma [2,36,37,39] or a specific setting where stigma is encountered (e.g., health care settings) [39,40], limiting their applicability. There have been efforts to develop stigma scales exclusive to PWID in other countries, but their generalizability to U.S. PWID has not been tested [8,41]. The result is that we lack a valid and reliable scale to assess drug-related stigma experienced by U.S. PWID across different settings, limiting our ability to comprehensively assess stigma and address stigma in this population.

Perhaps the most comprehensive of the existing drug-related stigma scales is the Substance Use Stigma Mechanisms Scale (SU-SMS) developed by Smith and colleagues [38], which was directly informed by the HIV Stigma Framework [33,34]. Smith’s 18-item scale (6 items each assessing enacted, anticipated, and internalized stigma, respectively) has high internal validity and reliability, as well as the ability to differentiate between these three distinct stigma mechanisms with respect to two separate sources (i.e., family and health care providers). In the decade since its introduction the SU-SMS has been employed by multiple studies [42–46] and validated in other countries [47–50]. However, its generalizability to stigma encountered by community-based PWID is likely limited because it was developed and validated in a sample consisting solely of in-care patients (methadone maintenance and individuals in HIV treatment), of whom only 9% had recently injected drugs. The sole focus on health care providers and family members is also limiting, as PWID are often marginalized from the health care system and estranged from family members.

Following the publication of the SU-SMS there have been additional efforts to develop a stigma scale specifically for PWID. For example, Fong et al. [40] developed and validated a scale to measure “medical provider stigma experience by people who use drugs” (MPS-PWUD) using a New York City based sample of community-recruited PWID but examined only enacted and internalized stigma in medical settings. Patel et al. [41] also used a community-based sample of PWID in 12 Indian cities to develop a more comprehensive drug use stigma scale addressing four types of stigma (enacted, internalized, vicarious, felt normative) in both health care and community settings, but included questions that are specific to Indian culture (e.g., “PWUD are paying for their karma or sins”); to the best of our knowledge, this scale has not been validated in a U.S. sample.

An additional limitation of these instruments is that they have been, without exception, developed in urban populations. This raises concerns regarding their generalizability to U.S. rural populations, who have experienced an unprecedented increase in injection drug use over the past decade [51,52] with accompanying risk of related infections [53,54]. Recent injection-associated HIV outbreaks and increases in injection-related endocarditis in rural communities [55–57] provide evidence of the need for stigma measurement instruments that have validity and reliability in rural settings. Recent studies have documented that drug-related stigma is a concern for PWID in non-urban communities due to its impact on health services access and utilization [58–60].

In this study, we sought to develop and validate a Drug Use Stigma Scale (DUSS) for community based PWID that can be used to assess multiple types of stigma across multiple settings. We were particularly interested in developing a scale that could help inform community intervention targets for stigma reduction. We recruited from two rural communities in West Virginia to ensure applicability to rural PWID populations.

## Materials and methods

Data collection for this study was conducted between May 23, 2023, and August 8, 2024. All research activities were approved by the West Virginia University Institutional Review Board (IRB) and all participants provided informed consent. To ensure anonymity for study participants, the WVU IRB approved a waiver of documented consent and related protocols for all phases for the study. For qualitative data collections, participants reviewed an IRB-approved consent form and provided verbal consent to participate. For quantitative data collection, participants reviewed an IRB-approved consent form as part of the computer-administered survey and indicated their consent by clicking on the appropriate response option before moving on to the survey instrument.

Scale development efforts progressed in phases with PWID input in each step of the development process. First, we used qualitative inquiry to adapt and expand scale items from the existing stigma literature. This involved in-depth interviews with PWID (N = 27) to augment questions from existing stigma scales, followed by three PWID focus groups (N = 28) designed to “member check” our interview findings to improve depth of knowledge and explore any additional areas of stigma lived experience. Based on this qualitative work and consultation with external experts we developed a preliminary 39-item stigma scale that was refined to a final 16-item scale (the DUSS) based on PWID survey responses (N = 336). More specific information on the methods involved in each of these data collection and analysis phases is provided in the following sections.

### Scale development

First, we established the following working definition of drug-related stigma in the community as after an extensive review of the literature: *Negative and unfair treatment that is perceived, anticipated, or experienced in community settings by those identified as using/injecting drugs. Considers both internal and external stigmatization associated with public settings, health care settings, drug treatment settings, family units, and drug using communities.*

Our aim was to develop a measure that captured a range of drug-related stigma perceptions and experiences, was valid and reliable among PWID, and could be tested in future research for use with individuals who use non-injection as well as injection drugs. We focused our development efforts on PWID, who experience the greatest degree of drug-related stigma and have been largely overlooked in previous research in this area.

Qualitative data collection and analysis. We used qualitative inquiry to adapt and expand scale items from the existing literature. Qualitative interview and focus group guides were aligned with the definition above to ensure representation of stigma types (anticipated, internalized, enacted) and settings (including family, society, and healthcare).

PWID who participated in qualitative interviews and focus groups were recruited in two West Virginia counties (“County A” and “County B”) using both direct recruitment by study staff at syringe services programs and peer referral. Eligibility requirements included being at least 18 years old and injecting illicit drugs in the past six months. Participants were reimbursed $40 for their time.

We conducted 27 interviews between May and October, 2023, exploring participants’ experiences with drug-related stigma in health care settings; in society more generally; and in interactions with family, police, and other people who use drugs. Interviews were recorded and transcribed verbatim. Based on careful reading of the transcripts we developed a preliminary coding scheme consisting of primary domains from the interview guide (deductive) and emergent themes (deductive), then developed a codebook from this coding scheme and further emergent themes. The codebook was piloted by two research assistants across four transcripts and the remaining transcripts were coded by both RAs, comparing coding for consistency and refining the codebook as necessary.

In May 2024 we convened three focus groups involving 28 PWID for the purpose of “member checking” [61,62] our qualitative interview findings. We presented each group with themes resulting from the qualitative interviews and asked for assistance in understanding these themes in more depth and identifying any experiences of drug-related stigma not already included in our analysis. Focus groups were recorded and transcribed and content was reviewed to inform the drafting of preliminary stigma scale questions.

Based on these efforts we developed a draft stigma scale that included selected questions from the literature (both verbatim and adapted) and new questions, consistent with our thematic analysis and member checking efforts.

PWID and subject matter expert review. We consulted both PWID and subject matter experts to refine the draft scale instrument. In June 2023, we conducted cognitive interviews [63,64] with four PWID to assess understanding and improve clarity of the scale items. Participants self-administered the draft scale and then were asked to discuss their overall impression of the instrument, their understanding of the questions, and the alignment of the questions with their own experiences of stigma. We also asked about their experience in reading and responding to the questions to detect any questions that might themselves be stigmatizing to PWID. The interviews were recorded and transcribed verbatim and then reviewed to identify areas for clarification and the need for additional scale items.

Also in June 2023, we distributed the draft scale to four external experts with experience conducting research on injection drug use and stigma. These experts provided feedback on question wording and clarity and recommended additional questions for inclusion.

After refinement we finalized the scale, which consisted of 39 items representing multiple stigma types (anticipated, internalized, and enacted) and relevant settings (family, society, and healthcare). All items were adapted to utilize a 5-point Likert-type response scale, including 1 “Strongly disagree”, 2 “Disagree”, 3 “Neither agree nor disagree”, 4 “Agree”, and 5 “Strongly agree”. Options were also given for “Not applicable” and “Refuse to answer”.

### Scale refinement

Survey recruitment and data collection. PWID recruitment was conducted in the same two West Virginia counties using the same methods, eligibility, and reimbursement as the scale development activities. Informed consent, screening questions, and survey were self-administered by participants on laptop computers using REDCap software [65,66]. The survey contained the 39-item stigma scale as well as questions on sociodemographics, drug use, health care experiences, HIV risk and services engagement, the HIV-related stigma scale [67], and additional questions regarding lived experience of stigma. We also included the SU-SMS questions [38] to facilitate post-hoc comparison with the newly developed scale. Two quality control items were included as well as a quality check of the inclusion criteria for injection drug use. These three variables were combined and used for sensitivity analysis in the final models.

Statistical analysis. All data management and analyses were conducted in SAS Version 9.4 [68] or Mplus Version 8.3 [69]. Continuous data are described using means and standard deviations, and categorical data are described using frequencies and valid percentages. Missing data, while minimal, was dealt with as appropriate for specific techniques, and included defaults of pairwise deletion or full information maximum likelihood (FIML) methods.

Item refinement. Stigma scale items went through a multi-dimensional refinement process prior to factor analysis on the development dataset. This approach was taken in response to i) a goal of eliminating unreliable or confusing items, and ii) an *a priori* desire to present a refined, quick screening tool of approximately 12–16 items. To be included in the final scale, each item was required to meet a series of three criteria: 1) Cronbach’s alpha for each item greater than or equal to 0.50, representing an acceptable degree of internal consistency; 2) a single factor Confirmatory Factor Analysis (CFA) beta greater than or equal to 0.60, indicating a moderate to strong relationship between the variable and the overall stigma construct assessed by the scale (i.e., the latent factor); and 3) items aligning with a three factor CFA based on stigma setting, wherein items coded by experts as having a stigma setting of “healthcare,” “society,” or “family” fell on their appropriate factors greater than or equal to 0.65. All CFAs were conducted on the polychoric correlations, given the ordinal nature of the items, using FIML method and setting the variances of the latent variables to 1.

Measurement CFA. Next, a series of CFA models were run first on the development sample (County A) and then the validation sample (County B). All CFAs were conducted on the polychoric correlations, given the ordinal nature of the items, using FIML method and setting the variances of the latent variables to 1. Models included 1) a single factor CFA of all items allowing stigma target errors to correlate, 2) a modified single factor CFA of all items and adding another error correlation, 3) three-factor CFA based on stigma setting (factors: healthcare, society, family) allowing latent factors to correlate. A final model was selected based on smallest Akaike information criterion (AIC) and model fit criteria that either were better or did not significantly decrease model fit (e.g., Root mean square error of approximation (RMSEA; preferred < 0.08), standardized root mean squared residual (SRMR; closer to 0 preferred), goodness-of-fit index (GFI; closer to 1 preferred), and Bentler-Bonett normed fit index (NNFI; closer to 1 preferred). Factor loadings for each model are given for the intercepts and slopes, and correlations for the errors allowed to correlate. Only the best fitting model is presented for the development and validation samples.

Validation. Two path analysis models (development, validation) were conducted to test criterion-related concurrent validation. These examined the average of the final set of items as correlated against similar stigma scales [38,40,67] and against a single item question of whether the individual did not seek healthcare even if they needed it (yes, no). The latter was included to evaluate the extent to which the full-scale score corresponds to a primary behavior of interest (healthcare avoidance), which previous literature suggests is strongly associated with drug-related stigma [4,6,7,18–21]. The models were adjusted for demographics that were hypothesized to be related to healthcare seeking behavior including gender and age. As the model had a binary outcome, a weighted least square estimator was used. Model fit criteria included RMSEA (preferred < 0.08), SRMR (closer to 0 preferred), and Comparative Fit Index (CFI) and Tucker-Lewis Index (TLI; preferred > 0.90). Factor loadings are presented for each item, and the loading against the binary outcome was exponentiated and presented as an odds ratio.

Sensitivity analysis. The final measurement model is presented for the combined sample of those from both development and validation groups who passed all three quality control measures to ensure that our model fit was appropriate against the most stringent sample.

## Results

Participants in the formative qualitative interviews (N = 27) and focus groups (N = 28) were majority female (59%) with a median age of 37 years and all but one (96%) were non-Hispanic White. Demographics of the survey participants, by group and as a total sample, are presented in Table 1. Across both samples there were 336 participants (development n = 216; validation n = 120). The majority of both samples were male (60% and 70% respectively) and were on average near 40 years of age. Similar to the qualitative sample, race and ethnicity characteristics were consistent with West Virginia’s state demographics: most participants were White (87% and 88%) and non-Hispanic (93% and 94%). Many of the participants reported experiencing homelessness (87% and 82%) and having Medicaid insurance (80% and 77%). Most participants in the development sample reported needing healthcare but not seeking it within the past 6 months (n = 140, 70%) compared to only about half of the validation sample (n = 57, 49%).

**Table 1:** Demographics for Development (n = 216), Validation (n = 120), and Total Sample (n = 336).

	Development Sample (n = 216)			Validation Sample (n = 120)			Total Sample (n = 336)		
Categorical Variable	*n*	*valid %*		*n*	*valid %*		*n*	*valid %*	
Gender									
Male	129	60		83	69.8		212	63.5	
Female	82	38.1		35	29.4		117	35	
Nonbinary/Other/Refuse	4	1.9		1	0.84		5	1.5	
Race									
White	187	87		105	88.2		292	87.4	
Black or African American	13	6.1		4	3.4		17	5.1	
All other/Refuse	15	7		10	8.4		25	7.5	
Ethnicity									
Non-Hispanic	199	92.6		112	94.1		311	93.1	
Hispanic/Latino	12	5.6		5	4.2		17	5.1	
Don’t know/Refuse	4	1.9		2	1.7		6	1.8	
Education									
Less than HS	42	19.5		33	27.7		75	22.5	
HS or GED	100	46.5		54	45.4		154	46.1	
Some college	66	30.7		30	25.2		96	28.7	
College or higher	4	1.9		2	1.7		6	1.8	
Don’t know/Refuse	3	1.4		0	0		3	0.9	
Current homeless/unstable housing									
Yes	172	87.3		89	82.4		261	85.6	
No	21	10.7		19	17.6		40	13.1	
Don’t know	4	2		0	0		4	1.3	
Insurance									
None	19	8.8		9	7.5		28	8.4	
Medicaid	171	79.5		91	76.5		262	78.4	
Medicare	18	8.37		13	10.9		31	9.3	
Other/Don’t know	7	3.3		6	5		13	3.9	
Employment									
Full-time	13	6.1		8	6.7		21	6.3	
Part-time/ Sometimes	50	23.3		37	31.1		87	26.1	
Disability	24	11.2		13	10.9		37	11.1	
Unemployed	122	56.7		58	48.7		180	53.9	
Other/Don’t know/Refuse	6	2.8		3	2.5		9	2.7	
Drugs injected past 6 months (check all)									
Heroin	163	75.5		74	61.7		237	70.5	
Fentanyl	166	76.9		85	70.8		251	74.7	
Cocaine	76	35.2		30	25		106	31.6	
Methamphetamine	166	76.9		88	73.3		254	75.6	
Suboxone or other prescription opioid	32	14.8		20	16.7		52	15.5	
Injection frequency past 6 months									
More than once a day	177	84.7		79	69.3		256	79.3	
Once a day	12	5.7		3	2.6		15	4.6	
2-6 days a week	11	5.3		19	16.7		30	9.3	
One day a week	4	1.9		3	2.6		7	2.2	
Less than once a week	2	1		8	7		10	3.1	
Don’t know/Refuse	3	1.4		2	1.8		5	1.6	
Ever been in drug treatment									
No	52	24.2		41	34.5		83	24.9	
Yes	161	74.9		78	65.6		249	74.6	
Don’t know/Refuse	2	0.9		0	0		2	0.6	
Needed healthcare but did not seek it									
Yes	140	69.7		57	49.1		197	62.2	
No	61	30.4		59	50.9		120	37.9	
**Continuous Variables**	***Mean (SD)***	***Median***	***25th, 75th***	***Mean (SD)***	***Median***	***25th, 75th***	***Mean (SD)***	***Median***	***25th, 75th***
Years since first injection									
Years	18.1 (10.9)	17	10, 25	16.7 (11.2)	14	8, 23	17.6 (11.0)	17	9, 25
Age									
Years	40.8 (9.1)	40	33.5, 47	39.5 (9.9)	39	32, 44	40.3 (9.4)	39	33, 46

### Item refinement

The development sample was used for initial item refinement. Table 2 includes all item questions, the source of each question, along with alignment to our definition including stigma type and intervention target. Sequentially working through the criteria for inclusion, 27 of the original 39 items met criteria 1 (internal reliability Cronbach alpha of 0.50 or greater). Next, a single factor CFA resulted in 22 items meeting the criteria of a beta of 0.55 or greater (criteria 2). The final criteria (criteria 3) examined a three-factor CFA of stigma setting (healthcare, society, and family). A total of 16 of the remaining 20 items met the inclusion criteria of a beta of 0.65 or greater. The final numbering of the items corresponds to intervention targets: Items 1–9 all target healthcare, items 10–13 society, and items 14–16 target the family (see Supporting Information S1 Appendix).

**Table 2:** Item refinement using Development Sample n = 216.

Item Question	Source	Stigma Type	Stigma Setting	Criteria 1. Alpha ≥ 0.5	Criteria 2. Single factor CFA beta ≥ 0.60	Criteria 3. Stigma based on setting beta ≥ 0.65		
						Healthcare	Society	Family
Healthcare workers have said critical or insulting things about my drug use	Fong 2021	Enacted	Healthcare	0.49				
**I have been treated disrespectfully by healthcare workers because of my drug use**	Adapted from Fong 2021	Enacted	Healthcare	0.56	0.69	0.74	0.00	0.00
If I was in recovery and relapsed, healthcare workers would criticize me for not trying harder	Adapted from Fong 2021	Anticipated	Healthcare	0.54	0.60	0.63	0.00	0.00
**I do not feel welcome in medical care settings**	Fong 2021	Enacted	Healthcare	0.58	0.73	0.79	0.00	0.00
When I go for medical care, I try to hide my drug use	Adapted from Fong 2021	Internalized or anticipated	Self, Healthcare	0.48				
When I go for medical care, I am embarrassed or ashamed about being a drug user	Adapted from Fong 2021	Internalized	Self, Healthcare	0.57	0.60	0.56	0.00	0.00
I do not want to disclose my drug use to healthcare workers	Fong 2021	Internalized or anticipated	Self, Healthcare	0.54	0.61	0.61	0.00	0.00
**I have been observed more closely than other patients in medical care settings because of my drug use**	NEW	Enacted	Healthcare	0.63	0.75	0.78	0.00	0.00
**I have been ignored by healthcare workers once they learn or suspect that I use drugs**	NEW	Enacted	Healthcare	0.72	0.83	0.86	0.00	0.00
**People who are known or suspected to use drugs are “red flagged” in the healthcare system**	NEW	Enacted	Healthcare	0.63	0.79	0.82	0.00	0.00
I avoid engaging in healthcare services related to my drug use because I might see someone I know	NEW	Internalized or anticipated	Healthcare	0.52	0.52			
**I am treated like a criminal in medical care settings because I use drugs**	NEW	Enacted	Healthcare	0.72	0.86	0.89	0.00	0.00
**Healthcare workers have thought that I’m pill shopping or trying to con them into giving me prescription medications to get high or sell**	Smith 2016	Enacted	Healthcare	0.63	0.74	0.74	0.00	0.00
**Healthcare workers have given me poor care because I use drugs**	Smith 2016	Enacted	Healthcare	0.66	0.82	0.85	0.00	0.00
**I have avoided getting health care because I expect I will be treated badly by healthcare workers**	NEW	Anticipated	Healthcare	0.70	0.81	0.81	0.00	0.00
I am comfortable advocating for myself in healthcare settings*	NEW	Internalized	Healthcare	0.03				
I have been threatened with physical harm because of my drug use	Adapted from Patel 2021	Enacted	Society	0.46				
My family has cut off contact with me because of my drug use	Adapted from Patel 2021	Enacted	Family	0.48				
**I will always be judged for my drug use even if I stop using drugs**	NEW	Anticipated	Self, Society	0.59	0.72	0.00	0.76	0.00
I am treated like a criminal by family members because of my drug use	NEW	Enacted	Family	0.48				
I have been denied or lost housing because of my drug use	Adapted from Patel 2021	Enacted	Society	0.55	0.54			
**People who experience homelessness are automatically assumed to be using drugs**	NEW	Anticipated or enacted	Self, Society	0.61	0.71	0.00	0.84	0.00
I have been denied or lost a job because of my drug use	NEW	Enacted	Society	0.51	0.52			
**I am treated like a criminal by society for using drugs**	NEW	Enacted	Self, Society	0.67	0.77	0.00	0.89	0.00
**I am watched more closely than other people in public places because of my drug use**	NEW	Enacted	Society	0.58	0.68	0.00	0.73	0.00
**I hide my drug use when interacting with my family**	NEW	Internalized or anticipated	Family	0.57	0.60	0.00	0.00	0.67
I hide my drug use when out in the community	NEW	Internalized or anticipated	Self, Society	0.54	0.59	0.00	0.58	0.00
I avoid services related to drug use because my family might find out that I use drugs	NEW	Anticipated	Family	0.51	0.46			
**I avoid my family because of my drug use**	NEW	Internalized or anticipated	Family	0.58	0.59	0.00	0.00	0.69
I hide my drug use because it would bring embarrassment or shame to my family	NEW	Anticipated	Family	0.57	0.57			
Family members do not trust me because of my drug use	Adapted from Smith 2016	Enacted	Family	0.52	0.56			
**Family members look down on me because of my drug use**	Adapted from Smith 2016	Enacted	Family	0.58	0.64	0.00	0.00	0.75
I am most comfortable with myself when I am around other people who use drugs*	NEW	Internalized	Society	0.48				
Mutual help meetings (like NA or AA) are not welcoming to people who are currently using drugs	NEW	Anticipated or enacted	Drug treatment/recovery, Family	0.34				
Family members of people who use drugs disapprove of medications for opioid use disorder (like methadone or buprenorphine)	NEW	Anticipated or enacted	Drug treatment/recovery, Family	0.43				
Healthcare workers disapprove of people who use medication for opioid use disorder (like methadone or buprenorphine)	NEW	Anticipated or enacted	Drug treatment/recovery, Healthcare	0.48				
The recovery community disapproves of people who use medications for opioid use disorder (like methadone or buprenorphine)	NEW	Anticipated or enacted	Drug treatment/recovery	0.39				
Society disapproves of people who use medications for opioid use disorder (like methadone or buprenorphine)	NEW	Anticipated or enacted	Drug treatment/recovery, Society	0.51	0.52			
I am concerned about being shamed by others for using medications for opioid use disorder (like methadone or buprenorphine)	NEW	Anticipated	Drug treatment/recovery	0.39				

*indicates reverse coded item; items in bold font retained in final scale.

### Measurement CFA

Next, the 16 DUSS items were included in a series of CFAs in which a single factor solution was included, paying particular attention to error correlations (by intervention target, and then a modification of the best fitting set or error terms). A three-factor solution plus modification were also used against intervention target. The best fitting model was a single factor solution allowing the errors of intervention target items to correlate with each other (all healthcare items were allowed to correlate, all society, and then all family) with an additional single error correlation allowed between two enacted society and family items [13 and 16]. Both development and validation sample results are included in Table 3, along with descriptive information about the scale and Cronbach’s alpha. For both models, means fell close to 4 on the 5-point scale (Ms = 3.78, 3.50) and Cronbach’s alpha were excellent (0.93, 0.95).

**Table 3:** Single factor CFA for development (n = 207) and Validation (n = 115) Samples, Allowing Correlated Errors by Intervention Target.

	Development Sample (n = 207)						Validation sample (n = 115)					
**Descriptive Statistics**												
DUSS mean (SD)	3.78 (0.82)						3.50 (0.98)					
Scale Cronbach’s alpha	0.93						0.95					
**Fit Summary**												
Fit Function	0.73						1.81					
Chi-Square	149.80						206.67					
Chi-Square DF	58						58					
Pr > Chi-Square	<.0001						<.0001					
SRMR	0.03						0.05					
GFI	0.92						0.84					
RMSEA (90% CL)	0.09	(0.07, 0.11)					0.15	(0.13, 0.17)				
Akaike Information Criterion	305.80						362.67					
CFI	0.96						0.92					
Bentler-Bonett NFI	0.94						0.90					
**Items**	***Unstandardized Estimate***	***SE***	***t Value***	***p-value***	***R-Square***	***Standardized Estimate***	***Unstandardized Estimate***	***SE***	***t Value***	***p-value***	***R-Square***	***Standardized Estimate***
1. I have been treated disrespectfully by healthcare workers because of my drug use	0.82	0.10	8.29	<.0001	0.33	0.57	0.89	0.12	7.60	<.0001	0.42	0.64
2. I do not feel welcome in medical care settings	0.76	0.08	8.96	<.0001	0.37	0.61	0.76	0.11	6.82	<.0001	0.35	0.59
3. I have been observed more closely than other patients in medical care settings because of my drug use	0.77	0.08	9.21	<.0001	0.39	0.63	0.83	0.12	7.18	<.0001	0.38	0.61
4. I have been ignored by healthcare workers once they learn or suspect that I use drugs	0.89	0.08	11.35	<.0001	0.54	0.73	0.96	0.11	8.79	<.0001	0.52	0.72
5. People who are known or suspected to use drugs are “red flagged” in the healthcare system	0.84	0.08	10.68	<.0001	0.49	0.70	1.03	0.11	9.54	<.0001	0.59	0.77
6. I am treated like a criminal in medical care settings because I use drugs	0.90	0.08	11.94	<.0001	0.58	0.76	0.95	0.11	9.01	<.0001	0.54	0.74
7. Healthcare workers have thought that I’m pill shopping or trying to con them into giving me prescription medications to get high or sell	0.82	0.08	9.86	<.0001	0.44	0.66	0.99	0.11	8.75	<.0001	0.52	0.72
8. Healthcare workers have given me poor care because I use drugs	0.91	0.08	11.90	<.0001	0.58	0.76	0.97	0.11	8.82	<.0001	0.52	0.72
9. I have avoided getting health care because I expect I will be treated badly by healthcare workers	0.87	0.07	11.71	<.0001	0.56	0.75	0.86	0.11	7.83	<.0001	0.44	0.66
10. I will always be judged for my drug use even if I stop using drugs	0.81	0.07	11.97	<.0001	0.59	0.77	1.10	0.11	9.91	<.0001	0.67	0.82
11. People who experience homelessness are automatically assumed to be using drugs	0.85	0.07	11.67	<.0001	0.57	0.76	1.00	0.10	10.46	<.0001	0.71	0.85
12. I am treated like a criminal by society for using drugs	0.84	0.06	13.02	<.0001	0.66	0.82	1.21	0.10	12.64	<.0001	0.88	0.94
13. I am watched more closely than other people in public places because of my drug use	0.70	0.06	10.93	<.0001	0.52	0.72	0.95	0.10	9.56	<.0001	0.64	0.80
14. I hide my drug use when interacting with my family	0.66	0.08	8.26	<.0001	0.32	0.56	0.89	0.10	8.95	<.0001	0.54	0.73
15. I avoid my family because of my drug use	0.67	0.08	8.30	<.0001	0.32	0.56	0.79	0.12	6.85	<.0001	0.35	0.59
16. Family members look down on me because of my drug use	0.66	0.06	10.49	<.0001	0.46	0.68	0.89	0.10	9.09	<.0001	0.55	0.74

Although both chi-squares were significant (expected given sample sizes) other model fit indicators ranged from good to excellent for both samples. For the development sample, fit statistics were all excellent, including SRMR: 0.03 and RMSEA: 0.09, both close to 0, and GFI: 0.92, CFI: 0.96, NFI: 0.94 all close to 1. Fit attenuated but remained satisfactory for the validation sample, with the GFI and RMSEA deviating from excellent fit (GFI: 0.84, RMSEA: 0.15), all other fit indicators remained excellent (SRMR: 0.05, CFI: 0.92, NFI: 0.90).

An examination of the specific item fit shows all items significantly related to the single factor. All standardized estimates were above 0.50 with most being above 0.61 across both samples, suggesting items can be used in conjunction with each other to assess our definition of stigma.

### Validation

Next, we examined our average scale scores as correlated with several related stigma scales and against the outcome of not seeking healthcare adjusting for full-time employment and gender (Table 4). Model fit for both the development and validation samples ranged from satisfactory to excellent. Both chi-squares were not statistically significant (p = 0.15, 0.06) as preferred. RMSEA were excellent (0.05, 0.08) and close to 0 as were the SRMR (0.05, 0.07). CFI were excellent for both models (0.98, 0.93). TLI was excellent for the development sample (0.95) but dropped slightly for the validation sample (0.84).

**Table 4:** Validation path analysis against related stigma scales and seeing healthcare for the DUSS development (n = 207) and validation samples (n = 120), adjusted for gender and age.

		Development Sample (n = 207)				Validation sample (n = 120)			
Descriptive statistics		*N*	*M*	*SD*		*N*	*M*	*SD*	
Smith stigma scale		208	3.5	0.8		117	3.2	1	
HIV stigma scale		175	2.9	0.7		116	2.7	0.8	
Fong stigma scale		213	3.5	1		119	3.3	1	
**Fit summary**		***Estimate***				***Estimate***			
Chi-Square		13.30				16.50			
Chi-square df		9				9			
Chi-square p-value		0.1494				0.0572			
RMSEA		0.05				0.08			
RMSEA 90% CL		0.000, 0.097				0.000, 0.146			
RMSEA p-value ≤ 0.05		0.48				0.17			
CFI		0.98				0.93			
TLI		0.95				0.84			
SRMR		0.05				0.07			
**Model Results**									
***Dependent Variable***	***Independent Variable***	***Estimate***	***S.E.***	***P-Value***	***OR***	***Estimate***	***S.E.***	***P-Value***	***OR***
Did not seek healthcare									
	NEW	0.39	0.11	0.001	1.47	0.48	0.11	<0.0001	1.61
	Male gender	−0.44	0.19	0.02	0.64	0.16	0.25	0.521	1.17
	Age	0.01	0.01	0.572	1.01	0.00	0.01	0.955	1.00
									
***Correlations***		***Estimate***	***S.E.***	***P-Value***		***Estimate***	***S.E.***	***P-Value***	
NEW	Smith stigma scale	0.41	0.06	<0.0001		0.74	0.12	<0.0001	
NEW	HIV stigma scale	0.20	0.04	<0.0001		0.34	0.07	<0.0001	
NEW	Fong stigma scale	0.58	0.07	<0.0001		0.78	0.14	<0.0001	
NEW	Age	−0.36	0.49	0.458		−1.75	0.96	0.07	
NEW	Male gender	0.00	0.02	0.881		−0.01	0.04	0.822	
Smith stigma scale	HIV stigma scale	0.31	0.04	<0.0001		0.29	0.06	<0.0001	
Smith stigma scale	Fong stigma scale	0.30	0.06	<0.0001		0.60	0.12	<0.0001	
HIV stigma scale	Fong stigma scale	0.16	0.04	<0.0001		0.31	0.07	<0.0001	
Male gender	Age	0.59	0.31	0.06		0.80	0.43	0.067	

For both models, the DUSS scores were significantly associated with increased odds of not seeking healthcare when needed (OR: 1.47, p = 0.001; OR: 1.61, p < 0.0001) after controlling for gender and age. Age was not associated with healthcare seeking, and male gender (as compared to all other genders) was only associated with healthcare seeking in the development sample.

The DUSS scores were also significantly associated with the other stigma scales. Specifically, scores were highly correlated with the Fong stigma scale [40] (Est: 0.58, 0.78), Smith stigma scale [38] (Est: 0.41, 0.74), and the HIV stigma scale [67] to a lesser degree, as anticipated (Est. 0.20, 0.34), all p < 0.0001.

### Sensitivity analysis

We combined our samples with only those who passed all three quality control questions with the correct answers and re-ran our single factor measurement CFA to ensure the model fit remained good-to-excellent. For this sample (n = 216) model fit remained excellent: SRMR and RMSEA both close to 0 (SRMR: 0.04, RMSEA: 0.09), and CFI, NFI, and GFI all near 1 (GFI: 0.92, NFI: 0.94, CFI: 0.96).

## Discussion

Drug-related stigma contributes to adverse health experiences and barriers to healthcare engagement among people who use drugs. Efforts to develop and evaluate interventions designed to reduce this stigma require valid stigma measurement instruments that can be applied across different stigma types, settings, and user groups.

In this study, we used both qualitative and quantitative methods to develop a new instrument, the DUSS, for assessing drug-related stigma among PWID. The instrument was developed and tested with participants from two rural samples and demonstrated high structural validity and internal reliability across multiple drug stigma mechanisms (anticipated, enacted, internalized) and settings (healthcare, family, society). This 16-item scale has several strengths to recommend its use over existing drug-related stigma scales.

First, our sample consisted of community recruited PWID. Some prior efforts have used treatment-based samples to facilitate study recruitment [2,35,36,38,70], but only a small percentage of people with substance use disorder actually receive treatment [71]. Accordingly, it is highly unlikely that scales developed using treatment-based samples are broadly generalizable to drug using populations. This is especially true in the context of stigma, which itself can serve as a barrier to treatment engagement [5,72].

Second, the DUSS was developed with input from, and in samples exclusive to, PWID. As noted, injection drug use is more highly stigmatized than other forms of drug use [1,2] and there is a growing literature specific to the experiences and impacts of stigma in PWID communities (e.g., 3, 4, 6, 7, 10, 11, 15, 18–21). Prior efforts among PWID were limited to non-U.S. based samples [8,41] or, for those conducted in the U.S., limited to stigma in healthcare settings [40]. The DUSS provides a tool for assessing stigma for U.S.-based PWID across multiple settings where stigma may be encountered. That said, we took care in drafting the wording of the scale items to ensure they could be easily applied to populations that use drugs by non-injection routes. Future studies should test the validity and reliability of the DUSS in these populations.

Third, our study is the first to validate a drug-related stigma scale in rural communities. While rural injection drug use, and drug use more broadly, has increased substantially in recent years, none of the existing stigma scales were developed or validated in rural populations. There is ample evidence to suggest that drug-related stigma persists in rural areas [58,59,73,74] and that people who use drugs in rural areas may encounter higher levels of stigma than those in urban areas [60,75]. Our formative qualitative work on this project elicited multiple comments regarding the challenges in maintaining anonymity as a person who uses drugs in small town settings compared to those in urban areas. Although our goal was to develop a scale that is relevant to the experiences of rural PWID, future studies should test the validity of the DUSS in both urban and suburban settings.

Fourth, the DUSS was developed with direct input from PWID, in contrast to efforts that have relied solely on literature review, theory, and/or researcher knowledge and expertise. As shown in Table 2, our qualitative work with PWID contributed several “new” questions to the scale that would have been missed had we only sought to validate existing stigma scales; indeed 11 of the items in our validated 16-item scale are “new” items that emerged from our qualitative work. These new items spanned multiple settings (healthcare, society/self, and family) and types of stigma (enacted, anticipated, internalized), emphasizing the breadth and depth of stigma experienced by PWID in their daily lives. Our reliance on the lived experience of PWID in developing our scale is consistent with more recent efforts to involve PWID in research and policy development that affects their lives and ensures that the DUSS captures those aspects of stigma that are most salient to this population.

It is important to note that our scale development and validation strategy prioritized qualitative findings over strict adherence to ensuring equal representation across various stigma domains (internalized, enacted, anticipated). Indeed, we found over the course of the study that many of the scale items developed during the qualitative phase did not fit neatly within a single stigma domain, which is perhaps not surprising given that these domains are theoretical. Again, we view this approach to item creation based on PWID’s lived experiences and input as a strength and note that the final validated scale still includes at least one item from each of three stigma domains.

Finally, the DUSS provides a scale that can be used to assess stigma across multiple settings. Unlike previous scales, this new scale addresses a broader range of settings (health care, family, society) corresponding to the lived experience of PWID. Our hope is that this will facilitate not just understanding of the nature of stigma experienced by PWID in each of these settings but prioritization of those settings where stigma reduction interventions are most warranted.

Our study does have limitations. The DUSS was developed based on data from PWID in a single rural state with limited racial and ethnic diversity and high levels (>80%) of homelessness and unstable housing. While we view the rural focus as filling an important gap in the literature, future studies should examine the validity of the scale in suburban and urban communities and more racially and ethnically diverse populations as well as other rural settings. Similarly, while we view our focus on PWID as a strength, future studies should examine the validity of the DUSS in other drug using populations.

## Conclusions

This study developed and validated a new drug-related stigma scale, the DUSS, that has several benefits over previously developed scales for assessing stigma among community based rural PWID. Although research is needed to test the validity of the scale in other settings and drug using populations, the DUSS provides a valuable tool for characterizing the nature and extent of stigma experienced by PWID and informing the development and testing of interventions designed to mitigate the impacts of drug-related stigma on their health and wellbeing.

## Supporting information

S1 Appendix Drug Use Stigma Scale (DUSS) Instrument and Scoring Instructions. (DOCX)
