# Supplementary material for: Drug-related stigma among people who inject drugs – development and validation of the drug use stigma scale (DUSS)
Source: PLoS One. 2025 Dec 12;20(12):e0338691. doi: 10.1371/journal.pone.0338691 (PMC12700462; doi:10.1371/journal.pone.0338691)
Supplement: S1 Appendix — (DOCX) [file pone.0338691.s001.docx]

**DRUG USE STIGMA SCALE (DUSS)**

Instructions: For each item below, please check (✓ ) the box that best answers how much you agree with the statement.

|  | (1)  Strongly disagree | (2)  Disagree | (3)  Neither agree nor disagree | (4)  Agree | (5)  Strongly agree |
| --- | --- | --- | --- | --- | --- |
| 1. I have been treated disrespectfully by healthcare workers because of my drug use | □ 1 | □ 2 | □ 3 | □ 4 | □ 5 |
| 1. I do not feel welcome in medical care settings | □ 1 | □ 2 | □ 3 | □ 4 | □ 5 |
| 1. I have been observed more closely than other patients in medical care settings because of my drug use | □ 1 | □ 2 | □ 3 | □ 4 | □ 5 |
| 1. I have been ignored by healthcare workers once they learn or suspect that I use drugs | □ 1 | □ 2 | □ 3 | □ 4 | □ 5 |
| 1. People who are known or suspected to use drugs are "red flagged" in the healthcare system | □ 1 | □ 2 | □ 3 | □ 4 | □ 5 |
| 1. I am treated like a criminal in medical care settings because I use drugs | □ 1 | □ 2 | □ 3 | □ 4 | □ 5 |
| 1. Healthcare workers have thought that I’m pill shopping or trying to con them into giving me prescription medications to get high or sell | □ 1 | □ 2 | □ 3 | □ 4 | □ 5 |
| 1. Healthcare workers have given me poor care because I use drugs | □ 1 | □ 2 | □ 3 | □ 4 | □ 5 |
| 1. I have avoided getting health care because I expect I will be treated badly by healthcare workers | □ 1 | □ 2 | □ 3 | □ 4 | □ 5 |
| 1. I will always be judged for my drug use even if I stop using drugs | □ 1 | □ 2 | □ 3 | □ 4 | □ 5 |
| 1. People who experience homelessness are automatically assumed to be using drugs | □ 1 | □ 2 | □ 3 | □ 4 | □ 5 |
| 1. I am treated like a criminal by society for using drugs | □ 1 | □ 2 | □ 3 | □ 4 | □ 5 |
| 1. I am watched more closely than other people in public places because of my drug use | □ 1 | □ 2 | □ 3 | □ 4 | □ 5 |
| 1. I hide my drug use when interacting with my family | □ 1 | □ 2 | □ 3 | □ 4 | □ 5 |
| 1. I avoid my family because of my drug use | □ 1 | □ 2 | □ 3 | □ 4 | □ 5 |
| 1. Family members look down on me because of my drug use | □ 1 | □ 2 | □ 3 | □ 4 | □ 5 |
| Column totals: |  |  |  |  |  |

Scoring Instructions:

Total average score: Sum of column totals, divided by 16

Subscale scores, representing average response for each intervention target:

1. Healthcare: Sum of items 1-9, divided by 9
2. Society: Sum of items 10-13, divided by 4
3. Family: Sum of items 14-16, divided by 3

Use of this scale:

This measure is freely available for use without permission so long as authorship is accurately attributed.
